# Seasonal Host Shifts for *Legionella* Within an Industrial Water‐Cooling System

**DOI:** 10.1111/1758-2229.70132

**Published:** 2025-06-30

**Authors:** Suzanne Crull, Emlyn Hammer, Allison E. Mann, Lauren M. O'Connell, Ashlyn Soule, Elizabeth Griffith, Thomas Blouin, Robin L. Brigmon, Vincent P. Richards

**Affiliations:** ^1^ Department of Biological Sciences Clemson University Clemson South Carolina USA; ^2^ Department of Anthropology University of Wyoming Laramie Wyoming USA; ^3^ Savannah River National Laboratory Environmental Science and Biotechnology Group Aiken South Carolina USA

**Keywords:** microbial communities, microbial ecology, pathogen ecology

## Abstract

*Legionella* is a genus of environmental bacteria containing pathogenic species such as *Legionella pneumophila* that are responsible for Legionnaires' disease, a potentially fatal respiratory infection. Disease aetiology can involve *Legionella* replication intracellularly within protists and this study aimed to characterise the *Legionella*‐protist relationship to develop novel outbreak prevention targets. Water and sediment samples were collected from a water‐cooling tower in South Carolina over a 6‐month period. Concomitantly, multiple environmental parameters were recorded. Bacterial and eukaryotic communities were characterised using 16S rRNA gene V4 region and a 252 bp fragment of 18S rRNA gene, respectively. Co‐occurrence network analyses were performed to elucidate *Legionella*‐protist correlations through time. We found that *Legionella* correlated with different protists as the seasons progressed. *Acanthamoeba* correlated with *Legionella* in early spring followed by *Vannella* and *Korotnevella* in late spring and early summer, and were joined by *Echinamoeba* in mid‐summer. *Vannella* and *Acanthamoeba* are known potential hosts for *Legionella*, while *Korotnevella* is a potential undocumented host. Of the environmental parameters, temperature showed strong correlation with protists genera, suggesting that *Legionella* abundance was driven by temperature‐dependent protist availability. Our results highlight ecological shifts that are associated with elevated *Legionella* levels, which offers potential targets to help predict and prevent disease outbreaks.

## Graphical abstract

Taxonomic profiling for *Legionella* (the causative agent of Legionnaires' disease) and protist hosts within a water cooling tower showed seasonal peaks for *Legionella* that were associated with taxonomic shifts in the protist community. *Legionella* frequency was tracked using amplicon sequencing (blue line) and serogroup profiling (red line). Protists were tracked using amplicon sequencing. Multiple environmental parameters were also profiled, and a strong correlation with temperature was observed, suggesting that *Legionella* frequency was driven by temperature‐dependent protist availability.

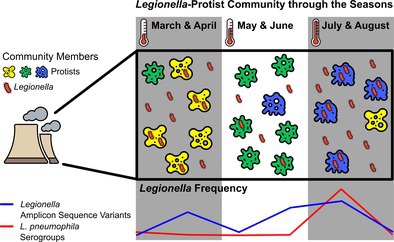

## 1 Introduction

*Legionella* is a genus of gram‐negative bacteria typically found in natural and engineered water systems as well as in soil (Fliermans et al. 1981; Wallis and Robinson 2005) that includes the species *Legionella pneumophila* , believed to be a major cause of Legionnaires' disease (Fraser et al. 1978; Sanden et al. 1992). Legionnaires' disease is a type of bacterial pneumonia, which includes symptoms such as fever, cough and chills (Kirby et al. 1980). In 2021, 50% of drinking water outbreaks and 60% of deaths associated with contaminated drinking‐water outbreaks in the United States were associated with *Legionella* (Centers for Disease Control and Prevention (CDC) 2024). Additionally, it is estimated that *Legionella* causes between 2% and 15% of all community‐acquired pneumonia that result in hospitalisation (Muder et al. 1989; Stout and Yu 1997). Along with Legionnaires' disease, *L. pneumophila* can cause Pontiac fever, a non‐pneumonic disease, which is a non‐fatal and less frequently reported infection that has a short incubation period with symptoms such as headaches, myalgia, fever and shivers (Cunha et al. 2016; Hamilton et al. 2018; Tossa et al. 2006). The exact number of Pontiac fever cases is hard to determine due to the lack of consensus on its definition and underreporting (Pancer and Stypułkowska‐Misiurewicz 2003; Tossa et al. 2006). Additionally, the exact number of Legionnaires' cases is unknown since an estimated less than 5% are reported (Cunha et al. 2016; Garrison et al. 2014; Marston and Breiman 1994). The majority of cases occur sporadically as well as lack of routine testing contributes to the underreporting of infections (Che et al. 2008; Marston and Breiman 1994; Moffa et al. 2023; Ricketts and Joseph 2005).

Legionnaires' disease is typically caused by the inhalation of aerosolized *Legionella* or aspiration of waterborne *Legionella*, which then infects the alveolar macrophages in the lungs using a similar mechanism to how *Legionella* infects protists (Fields 1996; Nash et al. 1984; Venezia et al. 1994). *L. pneumophila* is divided into at least 17 serogroups, which can be detected using monoclonal antibodies since serogroups are based on different surface antigens, such as outer membrane proteins and lipopolysaccharides (Helbig et al. 2002, 1997). Serogroup one is detected in more than 80% of cases caused by *L. pneumophila* during Legionnaires' disease outbreaks, while serogroup six is the second most common cause (Katsiaflaka et al. 2016; Ratcliff et al. 1998; Yu et al. 2002). Additionally, serogroups two through eight and 13 have also been isolated from Legionaries' disease cases as well (Yu et al. 2002). Other species of *Legionella* that are known to cause Legionnaires' disease include *Legionella longbeachae* , *Legionella micdadei* , *Legionella bozemanae* and *Legionella dumoffi* (Cunha et al. 2016; Marston and Breiman 1994; Ricketts and Joseph 2005; Yu et al. 2002). Exposure to *Legionella* is associated with different sources of infection (Hines et al. 2014) including showers (Bollin et al. 1985), faucets (Bollin et al. 1985), toilets, humidifiers (Arnow et al. 1982), whirlpool spas (Armstrong and Haas 2008; Jernigan et al. 1996), hot springs (Armstrong and Haas 2008), water‐wall‐type decorative fountains (Haupt et al. 2012), potting soil (Duchin et al. 2000; Ruehlemann and Crawford 1996; Wallis and Robinson 2005), and cooling towers. In particular, cooling towers, devices used to cool buildings and industrial processes, can aerosolize water, which has often been linked to outbreaks of Legionnaires' disease, and previous studies have detected the presence of *Legionella* within such towers (Bentham and Broadbent 1993; Bhopal et al. 1991; Brown et al. 1999; Hamilton et al. 2018; Ishimatsu et al. 2001). Namely, approximately 50% of confirmed Legionnaires' disease outbreaks and 60% of deaths from 2006 to 2017 were associated with cooling towers (Hamilton et al. 2018). Cooling towers have also been linked to sporadic cases of Legionnaires' disease (Bhopal et al. 1991; Hamilton et al. 2018). Conditions within cooling towers provide an optimal environment for *Legionella* proliferation due to a combination of elevated water temperatures, water stagnation and the accumulation of sediment (Centers for Disease Control and Prevention (CDC) 2024; Ciesielski et al. 1984; Kusnetsov et al. 1996; Stout et al. 1985).

Legionnaires' disease outbreaks are associated with certain environmental factors that increase the frequency of *Legionella*, including increased temperature (Kusnetsov et al. 1996), water stagnation (Ciesielski et al. 1984) and the presence of certain protists. Inclement weather has also been linked with increased *Legionella* densities in cooling tower water at the Savannah River Site, the same location our samples were collected from, due to an increase of debris entering the system (Brigmon et al. 2020). The presence of protist hosts (i.e., protists that consume but cannot digest *Legionella*) provide refuge for *Legionella*. Within the protists, *Legionella* can replicate, resulting in an increased concentration of *Legionella* and heightened risks of Legionnaires' disease (Barbaree et al. 1986; Emmerson 2001; Sanden et al. 1992; Shelton et al. 1994). Typically, *Legionella* are ingested by protists and infect their vacuoles, where they replicate until the cell is killed and lysed (Rowbotham 1980). Other times, infection is not confined to the vacuole, and the protist transforms into a vessel housing non‐motile Legionella, and as the concentration of Legionella increases, the protist loses its cellular infrastructure and is more likely to lyse and release Legionella into the environment (Newsome et al. 1998; Rowbotham 1980). Furthermore, cultured water tower samples containing amoebae resulted in more *Legionella* than samples lacking amoebae (Sanden et al. 1992).

In 1980, *Legionella*'s ability to replicate inside of amoeba was first observed (Rowbotham 1980) and since then, further studies have identified more host amoeba and host ciliates that support *Legionella* replication (Fields et al. 1984). The phyla Amoebozoa, Ciliophora and Percolozoa (Boamah et al. 2017; Gast et al. 2011; Nisar et al. 2020) contain certain protist genera that are likely environmental hosts for *Legionella*, such as *Acanthamoeba* (Molmeret et al. 2005; Rowbotham 1980, 1983), *Cyclidium* (Barbaree et al. 1986), *Echinamoeba* (Fields et al. 1989), *Naegleria* (Baldassarre et al. 2013; Rowbotham 1980), *Tetrahymena* (Barbaree et al. 1986), *Vannella* (Gast et al. 2011; Kuroki et al. 1998; Nisar et al. 2020) and *Vermamoeba* (formally *Hartmenella* (Smirnov et al. 2011)) (Brieland et al. 1996; Fields et al. 1989; Rowbotham 1986). Specifically, *Legionella* has been observed in environmentally isolated protists such as *Acanthamoeba*, *Vannella* and *Vermamoeba* (Declerck, Behets, van Hoef, et al. 2007; Gast et al. 2011; Kurtz et al. 1982). Additionally, *Acanthamoeba*, *Echinamoeba*, *Vannella* and *Vermamoeba* have been reported as common taxa in water‐cooling towers (Paniagua et al. 2020; Paranjape et al. 2020; Pinel et al. 2021; Tsao et al. 2019). Conversely, the phyla Cercozoa and certain species of the genus *Vannella* seem better able to resist *Legionella* infection through either digestion (*Legionella* predators) or potentially avoiding consumption of *Legionella* (Amaro et al. 2015; Boamah et al. 2017; Gast et al. 2011; Rowbotham 1983, 1986).

Protists not only provide a location for *Legionella* replication but also confer other benefits to the bacteria. For example, *L. pneumophila* acquire increased resistance to chemical biocides (Barker et al. 1992; Berk et al. 1998; Casini et al. 2018), antibiotics (Barker et al. 1995) and chlorine (Cervero‐Aragó et al. 2015; Kilvington and Price 1990) after replicating in protist hosts. Proposed explanations for this increased resistance include the protist hosts acting as a shield from the environment, as well as phenotypic changes in *Legionella* that include decreased size and increased motility after replication inside of amoebae, making the bacteria more resistant to chemical treatments (Barker et al. 1992, 1995). In addition to imparting increased resistance to disinfection techniques, residing inside a protist allows *Legionella* to recolonize a water system after the system was cleared of free‐living *Legionella* since *Legionella* is able to hide in the protist (García et al. 2007; Taylor et al. 2009; Thomas et al. 2004). Additionally, protists increase the infectivity (Cirillo et al. 1994, 1999) and severity of *L. pneumophila* infections in mice (Brieland et al. 1996). The increased infectivity is possibly due to different mechanisms of uptake, phenotypic changes for *Legionella* and different gene expression during entry (Brieland et al. 1996; Cirillo et al. 1994, 1999).

Previous research on the microbiome of cooling towers has demonstrated that the structure of bacterial and protist communities is highly dynamic, with environmental factors impacting the composition of the microbial community, which in turn impacts the ecology of *L. pneumophila* (Paniagua et al. 2020; Paranjape et al. 2020; Pinel et al. 2021; Tsao et al. 2019). Previous longitudinal studies have focused on inter‐kingdom microbiome interactions and have shown that while the microbiome is highly dynamic, core communities are biofilm‐forming, with inter‐kingdom relationships playing a key role, particularly since evidence suggests *L. pneumophila* grows within biofilm‐associated host cells before being released into the water following host cell lysis (Paniagua et al. 2020; Tsao et al. 2019). In addition, environmental factors such as temperature, dissolved organic carbon and pH have been shown to impact the bacterial and protist community composition as well as the frequency of *Legionella* (Paniagua et al. 2020; Paranjape et al. 2020; Pinel et al. 2021). Despite these findings, there has been no investigation into the role that environmental change might have in shaping *Legionella*'s associations with protists through time.

Here, over a six‐month period, we focused on an industrial water‐cooling tower known to be colonised with *L. pneumophila* . We combined 16S rRNA and 18S rRNA amplicon sequencing with network analysis to identify correlations between *Legionella* and protists and characterise this dynamic through time. Results showed strong transitions in abundance for different potential protist host genera through the seasons coupled with apparent host switching for *Legionella* that likely facilitated its persistence. Identifying the protists that facilitate *Legionella*'s survival in water systems will enhance our knowledge of Legionnaires' disease and provide potential targets to help reduce *Legionella* prevalence, as traditional approaches are less effective against protist‐associated *Legionella*.

## 2 Materials and Methods

### 2.1 Water Cooling Tower Sample Collection and Measurement of Environmental Parameters

We collected water samples as well as sedimentation exposed to sunlight and sedimentation not exposed to sunlight from the basin of a water‐cooling tower (HTF‐2) known to be colonised with *L. pneumophila* , which was located at the Savannah River National Laboratory in Aiken, South Carolina. The sediment samples, particulates from the bottom of the basin, were collected using a 50‐mL syringe attached to polycarbonate tubing. The tubing and syringe were rinsed 3X with basin water before pulling the sediment sample. HTF‐2 is a Marley three‐cell tower with crossflow induced‐draft unit featuring galvanised steel upper works and stainless‐steel components. It is mounted on a concrete basin and equipped with plastic drift eliminators. The system has a maximum flow capacity of 9000 gal per minute (3000 GPM per cell). Water samples were screened for the presence of *L. pneumophila* serogroups one, two, four and six using Direct Fluorescent Antibody (DFA) testing. HTF‐2 has a sister tower (HTF‐1) that operates when HTF‐2 is closed for cleaning. HTF‐2 was chosen since it had the longest time of operation between the towers for a total of 21 weeks before it was shut off for cleaning. Sampling for HTF‐2 began when HTF‐1 was turned off for cleaning and HTF‐2 was turned on for operation. Samples were collected on a near‐weekly basis (March 15th, 2016 through August 3rd, 2016). A total of 47 samples were collected as follows: 15 water samples, 17 dark sediment samples and 15 bright sediment samples (Table S1). We also measured seven environmental parameters monthly: temperature (°C), dissolved oxygen (DO) (%), conductivity (Siemens per metre [S/m] in SI and millimhos per centimetre [mmho/cm]), pH, free chlorine concentration, bromide concentration and turbidity. A calibrated Yellow Springs Instrument (YSI) MPS 552 multi‐parameter metre was used to measure temperature, DO, conductivity and pH. Palintest 1000 test kits were used to measure the total Br and/or free Cl. DFA for *L. pneumophila* serogroup one, two, four and six was also performed monthly on HTF‐2 over the sampling period, which gave cells/L for the serotypes. During the sampling period, ChemTreat CL‐49, a specialised industrial biocide water treatment containing both chlorine and bromide, was added continuously to the tower for a target concentration of 0.2–0.5 ppm. A corrosion inhibitor, Drew 2235 Cooling Water Treatment, was also added at a recommended level of 0.7–1.1 ppm to reduce the corrosive effects of ChemTreat.

### 2.2 Direct Fluorescent Antibody Testing

DFA testing was conducted monthly for *L. pneumophila* serotypes one, two, four and six since those serotypes have been previously linked to disease (Shelton et al. 1994). We first filtered water samples through a membrane (47‐mm‐diameter, 0.4‐μm pore size) prior to DFA testing. We then aseptically cut and placed the filters into a 15 mL conical tube before adding 1 mL of 0.2‐μm‐filter sterilised FA Buffer. The tube was then vortexed for 4 min. We pretreated eight‐well glass slides at 90°C and rinsed with 70% ethanol prior to use. For a positive control, we used inactivated *L. pneumophila* serogroups one, two, four and six cells from Monoclonal Technologies Inc. (Alpharetta, GA, USA). For the negative control, we used *Serratia marcescens* (ATCC 13880) and water as a test for non‐specific binding. We then added sample replicates (10 μL) to four wells of the prepared slide and heat fixed for 10–15 min at 80°C–90°C. Next, we placed the slides into a 25°C humidified chamber for 20 min before staining the slides separately with 20 μL of antibody fluorescein isothio‐cyanate (FITC)‐labelled affinity purified polyclonal antibodies for *L. pneumophila* serogroups one, two, four and six (Monoclonal Technologies Inc). We then rinsed the slides with DI water, which were then stored overnight in FA buffer at 25°C. Afterward, we rinsed the slides with 5% sodium pyrophosphate buffer and dried them in the dark at 25°C. We then examined the slides and counted the DFA labelled *L. pneumophila* cells with fluorescent microscopy (Zeiss Axioscope 2) at 1000×. Concentrations of the species and serotype were determined as previously described (Leskinen et al. 2012). Previous studies have delineated concentrations of *Legionella* (cells/L) into three categories: health threatening (10^6^ cells/L), high *Legionella* concentration (10^7^ cells/L) and high level of concern (10^8^–10^9^ cells/L) (Atlas 1999; Reinthaler et al. 1988; Shearer 1996). Here, using combined *L. pneumophila* serogroup counts, we categorise the concentrations as green (0–10^6^ cells/L), yellow (10^6^–10^7^ cells/L) and red (10^7^ cells/L or greater). In July, the tower was ‘red’ and required biocide shocking treatment to bring *L. pneumophila* concentrations down.

### 2.3 DNA Extraction

We extracted DNA from the sediment and water samples using the boil method as described previously with slight modifications from the protocol described in Peng et al. (2013) and Yamagishi et al. (2016). We added 0.5% Tween 20, a detergent that permeabilizes the cell membranes, and an even mix of 0.1 and 0.5 mm glass beads (Fisher Scientific) to aliquots of the samples and mechanically disrupted them in a bead beater for 5 min. Next, the samples were boiled for 10 min at 100°C to further break open the cells. We then centrifuges the boiled samples for 10 min at 10,000 g to separate the cell debris from the DNA in the supernatant. The supernatant was stored at −20°C until DNA sequencing library construction.

### 2.4 Illumina Library Construction and Sequencing

To profile the prokaryotic community, we used the 16S rRNA gene V4 region, which was amplified using universal bacterial primers from Kozich et al. (2013). The forward primer was 515f, and the reverse primer was 806r (Caporaso et al. 2011). We designed custom 18S rRNA protist PCR primers using a global nucleotide alignment that contained protist genera commonly known to be associated with *Legionella*. Specifically, the alignment included a total of 23 protist sequences, with two sequences from *Acanthamoeba*, two from *Balamuthia*, one from *Diphylleia*, four from *Echinamoeba*, four from *Vermamoeba*, one from *Naegleria*, two from *Neoparamoeba*, three from *Saccamoeba*, two from *Vahlkampfia* and two from *Vannella* (Table S2). Primer sequences were as follows: 18S rRNA FWD 5′‐AGAYGATYAGATACCGTCGTAG‐3′ (22 bp with 2 degenerate nucleotides), 18S rRNA REV 5′‐GGTGYCCYTCCGTCAATTCCTTT‐3′ (23 bp with 2 degenerate nucleotides). The target amplicon began at position 1365 bp of the 18S rRNA gene alignment and because of size variation among taxa, ranged in length from 148 to 252 bp. The custom primers captured a broad taxonomic range in our samples with 46 phyla taxonomically assigned, encompassing protists, fungi, plants and metazoans (Figure S1).

We constructed the 16S rRNA and 18S rRNA amplicon libraries in a single PCR using the primers described above and 17 μL of AccuPrime Pfx Supermix, 1 μL of DNA, 1 μL of forward primer and 1 μL of reverse primer. Thermocycler conditions were 95°C for 2 min, followed by 31 cycles of 95°C for 20 s, 55°C for 15 s and 72°C for 5 min. This was followed by a final elongation step of 72°C for 10 min. The libraries were then quantified using a Qubit 2.0 Fluorometer (Invitrogen) and pooled at an equimolar concentration of 2 nM. We sequenced the libraries on the Illumina MiSeq platform using V2 chemistry (250 bp, PE reads).

### 2.5 Sequence Processing

Qiime2 v2023.2.0 (Bolyen et al. 2019) was used to analyse 16S rRNA and 18S rRNA amplicon data. The demultiplexed forward and reverse reads were imported into Qiime2. The sequenced region of 18S rRNA showed considerable length variation among taxa from 85 to 219 bp. Cutadapt v4.2 (Martin 2011) was used to remove any contaminating primer or adaptor sequence in the 16S and 18S rRNA sequences. Next, we used the DADA2 v1.26.0 plugin (Callahan et al. 2016) to remove chimeric and low‐quality sequences and generate amplicon sequence variants (ASVs). We classified the 16S rRNA ASVs using the classify‐sklearn naïve Bayes taxonomy classifier (Bokulich et al. 2018) from scikit‐learn v0.24.1 using the EzBioCloud database (Yoon et al. 2017) as reference. Using the Silva database v. 138 (Glöckner et al. 2017; Quast et al. 2013; Robeson et al. 2020), we classified the 18S rRNA ASVs. For the 16S rRNA dataset, we removed eukaryote‐assigned taxa and bacteria that did not have phyla assigned. For the 18S rRNA dataset, we removed all ASVs not assigned at the phyla level. Rarefaction without replacement was then conducted at a depth of 13,430 and 7445 reads for the 16S rRNA samples and 18S rRNA samples, respectively. Rarefaction depth was chosen as 99% of the minimum sample depth. Alpha rarefaction curves were generated for the respective depths to ensure the full diversity was captured (Figure S2). The data was then exported into R v4.2.3 (R Core Team, n.d.) and CoNet v1.1.1 beta (Faust and Raes 2016; Hu et al. 2018) for diversity and correlation analyses. All three sample categories (water, dark sediment and bright sediment) were grouped and analysed together for downstream analysis since there were no significant differences in beta diversity among the categories for either the prokaryotic or eukaryotic communities. Beta diversity was calculated using the Bray–Curtis dissimilarity, and significance was assessed using PERMANOVA analysis on the rarefied data.

### 2.6 16S rRNA and 18S rRNA Relative Abundance Analysis

First, to examine overall changes in the prokaryotic and eukaryotic communities, we made phyla‐level relative abundance plots for each month between March 2016 and August 2016 and by individual samples for both the 16S rRNA and 18S rRNA communities using the R libraries tidyverse v.2.0.0 (Wickham et al. 2019), phyloseq v1.24.0 (McMurdie and Holmes 2013) and scales 1.2.1 (Hadley and Seidel 2022). We converted the ASV counts into relative abundance using microbiome v1.21.1 (Lahti and Shetty 2017). Next, we collapsed all phyla together that were less than 1% of the population into a single category and visualised the data using ggplot2 v3.4.1 (Wickham 2016).

To investigate the relationship between *Legionella* and its potential hosts and predators, we filtered the 18S rRNA ASVs to only contain the phyla Amoebozoa, Ciliophora, Heterolobosea and Cercozoa. These phyla contained species that have been previously identified to either be potential *Legionella* hosts or predators, as seen in Table S3. We classified Amoebozoa, Ciliophora and Heterolobosea as potential host phyla and Cercozoa as a potential predator phylum.

We then made a monthly relative abundance plot of the genera in the phyla that contained previously identified hosts or predators and overlaid the relative abundance of *Legionella* and the concentration (cells/L of water) of total *L. pneumophila* SGs. Phyloseq was used to filter out the phyla that were not potential hosts or predators of *Legionella* and collapse the ASVs by genera. The R library microbiome was used to calculate the relative abundance. We obtained *Legionella* relative abundance by collapsing all bacteria down to the genus level and transforming the counts into relative abundance using the microbiome package. We then removed all non‐*Legionella* bacteria using phyloseq. We used ggplot2 and ggforce v0.4.1.9000 (Pedersen 2022) to visualise the relative abundance for the targeted protist phyla, the relative abundance of *Legionella* and the total SG density of *L. pneumophila* .

### 2.7 Environmental Correlation

Possible correlation between environmental parameters and alpha diversity were explored using the rarefied samples and the Hmisc v5.0‐1 (Harrell and Dupont 2023) R library to calculate the Spearman correlation (Spearman 1904). To account for multiple hypothesis testing, we adjusted the *p*‐values using the Benjamini–Hochberg procedure (Benjamini and Hochberg 1995) in the R library tabletools v0.1.0 (Luther 2023). After correction, *p*‐values less than 0.05 were considered significant.

To detect environmental parameters that correlate with increased or decreased community dissimilarity, we calculated Spearman correlations between beta diversity and environmental samples on centered log‐ratio (CLR) transformed abundance for both the 16S rRNA and 18S rRNA ASVs. CLR transformation is a log transformation that normalises the data into a form of relative abundance that accounts for the complex compositional data structure of microbiome data, and therefore reduces the likelihood of spurious correlations (Gloor et al. 2016; Nearing et al. 2022). The R library vegan 2.6‐5 (Oksanen et al. 2023) was used to calculate the Euclidean distance for both the community and environmental matrix as well as the Spearman correlation between the environment and beta diversity.

Using the Spearman Rank coefficient, the correlation between environmental parameters and (i) *Legionella* ASV frequency and (ii) serogroup (cells/L of water) respectively was assessed within R. We used microbiome v1.21.1 to convert the data into relative abundance. Hmisc v5.0.1 was used to calculate the Spearman correlation. We only kept *p*‐values that were less than 0.05 after adjustment using the Benjamini–Hochberg procedure in the R library tabletools v0.1.0. Additionally, this analysis was repeated using average daily temperature for the sampling date from the weather station at Augusta Regional at Bush Field, located approximately 14 miles from SRNL, but no further correlations were identified.

### 2.8 Network Analysis

To determine how *Legionella* and protist taxa were correlated, we constructed networks using CoNet and Pearson correlation at the ASV level. We used CoNet version 1.1.1 beta (Faust and Raes 2016; Hu et al. 2018) and Pearson correlation (Pearson 1909) to generate association networks. All eukaryotic ASVs that did not belong to the previously identified host and predator phyla (Table S3) and all 16S rRNA ASVs that were not identified as *Legionella* at the genus level were removed. Samples were grouped into bimonthly categories because (i) there was no significant difference in *Legionella* abundance from March to April, May to June and July to August (Figure S3) and (ii) there was no significant difference in beta diversity between either the bacterial or eukaryotic communities within each two‐month period. This grouping increased the number of data points and consequently statistical power for the analyses. We used Kullback–Leibler dissimilarity (Kullback and Leibler 1951), Bray–Curtis dissimilarity (Bray and Curtis 1957), Spearman correlation and Pearson correlation (Pearson 1909) to construct the CoNet network in Cytoscape version 3.9.1 (Shannon et al. 2003). Kullback–Leibler and Bray–Curtis were used since they are robust to compositionally and matching zeroes (Chen et al. 2021; Shannon et al. 2003). Spearman was used since it is robust to outliers (Faust and Raes 2016; Hu et al. 2018). Permutation and bootstrap distributions were generated for the top 1000 correlations that had the most strength. The dissimilarity and correlation specific *p*‐values were computed as the area of the mean of the permutation distribution under the Gauss curve generated from the mean and standard deviation of the bootstrap distribution. The *p*‐values were merged using Brown's method (Brown 1975) and adjusted using the Benjamini–Hochberg procedure (Benjamini and Hochberg 1995). The significant edge (*α* < 0.05) table was saved and exported. The edge table network was visualised in R version 4.2.3 (R Core Team 2023) using the package circlize v0.4.15 (Gu et al. 2014) to make chord diagrams.

For the Pearson correlation, we first converted the 16S rRNA and 18S rRNA ASV counts into relative abundance using the R library microbiome v1.21.1 (Lahti and Shetty 2017). We then calculated the Pearson correlation using the R library Hmisc v5.0.1 with the *p*‐value adjusted using the Benjamini–Hochberg procedure. Adjusted *p*‐values that were less than 0.05 were kept for visualisation using the R library circlize v0.4.15.

### 2.9 Protist Host Abundance Through the Seasons

For the four genera that had of 5% or more of the connections with *Legionella* in correlation networks (*Acanthamoeba*, *Echinamoeba*, *Korotenevella* and *Vannella*), we visualised their frequency distributions through the seasons within a phylogenetic framework as follows. Phylogenetic reconstruction followed the Eukref pipeline (del Campo et al. 2018). 18S rRNA reference sequences were obtained from the PR2 database v5.0.0 (Guillou et al. 2013), Silva release 138 database (Glöckner et al. 2017; Quast et al. 2013; Robeson et al. 2020) and NCBI Nucleotide database (Sayers et al. 2022). We used vsearch v2.15.1 (Rognes et al. 2016) to cluster the reference sequences at 99% identity and aligned the clusters using MAFFT v7.475 (Katoh and Standley 2013). We then used trimAL v1.2 (Capella‐Gutiérrez et al. 2009) to trim the alignment and RAxML v8.2.12 (Stamatakis 2014) to construct a reference phylogeny using the substitution model GTRCAT.

Next, 18S rRNA ASV sequences were aligned to the reference 18S rRNA alignment using SINA v1.7.1 (Pruesse et al. 2012). The resulting alignment was used to construct a final phylogeny using RAxML with the reference phylogeny as a constraint. Archaeopteryx 0.968 beta (Zmasek, n.d.) was used to remove any large lineages that did not contain ASVs. We used FigTree v1.4.4 (Rambaut 2010) to visualise the phylogeny.

We generated heat maps for CLR transformed abundance for the targeted protist ASVs by month and overlaid theses on the final phylogeny using the R libraries phyloseq v1.24.0, RColorBrewer v1.1.3 (Neuwirth 2022), tidyverse v2.0.0, microbiome v1.21.1, reshape2 v1.4.4 (Wickham 2007) and ape v5.7.1 (Paradis and Schliep 2019) to construct the heatmaps. To compare CLR‐transformed abundances across the three bimonthly groups, we used boxplots and Wilcoxon tests, adjusting *p*‐values for multiple comparisons using the false discovery rate. Analyses were conducted using phyloseq v1.24.0, RColorBrewer v1.1.3, tidyverse v2.0.0, microbiome v1.21.1, reshape2 v1.4.4, ape v5.7.1, ggpubr v0.60 (Kassambara 2023b) and restatix v0.7.2 (Kassambara 2023a) to create the boxplots.

## 3 Results

### 3.1 Shifts in *Legionella* Abundance Correlated With Different Host Abundance

For all samples combined, there were a total of 1581 18S rRNA ASVs and 2351 16S rRNA ASVs that passed filtering steps. In total, 70 of the 16S rRNA ASVs were assigned to *Legionella*. Of the 70 ASVs assigned to the genus Legionella, only five species were assigned ( *Legionella shakespearei* , *Legionella quinlivanii* , *Legionella geestiana* , *Legionella drozanskii* and *Legionella beliardensis* ), while the remaining ASVs were classified only to the genus level. On average, there were 98 18S rRNA ASVs and 159 16S rRNA ASVs per sample. For both the eukaryotic and bacterial beta‐diversity, there were no significant bimonthly changes. There were no significant bimonthly changes in eukaryotic alpha diversity. For bacterial alpha diversity, March/April was significantly lower than both May/June and July/August. *L. pneumophila* serogroups were consistently detected over the 6‐month period.

**FIGURE 1:**
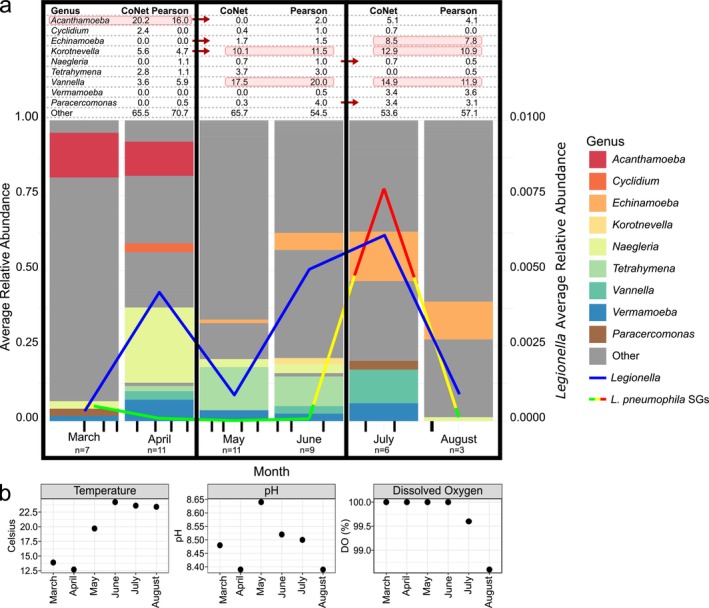
(a) *Legionella* and protist genera average relative abundance through time. *Legionella* (16S rRNA amplicon frequency data) is represented by the blue line overlayed on the bar graph. *L. pneumophila* serogroups (SGs) (cells/L) are represented by the contiguous green, yellow and red line. Colour indicates *L. pneumophila* concentration (green: 0–10^6^ cells/L, yellow: 10^6^–10^7^ cells/L, red: 10^7^ cells/L or greater). In the bar chart, protist genera possessing species known to interact with Legionella are coloured. *Korotnevella* was also coloured due the comparable number of correlations with Legionella compared to the protist hosts with the most correlations. Remaining genera belonging to the protist phyla Amoebozoa, Cercozoa, Ciliophora and Heterolobosea are categorised as ‘Other’ and coloured grey. The table represents the proportion of positive ASV correlations between *Legionella* and the specified protist genus out of the total *Legionella*‐protist correlations for the bimonthly networks (CoNet and Pearson approaches) (high proportions are highlighted in red). Arrows indicate a statistically significant difference in log‐transformed abundance between the bimonthly categories. Sampling dates for each month are shown at the bottom of each bar and are indicated by vertical black lines. (b) Changes in temperature, pH and dissolved oxygen by month for the water‐cooling tower.

Overall, we detected large seasonal shifts in abundance for all protist host genera (Figures 1a and S4). We also detected large swings in abundance for *Legionella*, with periods of high abundance correlated with different host protists as the seasons progressed (Figure 1a). Just under half of *Legionella*'s correlations were with genera that were previously identified to contain host species for *Legionella*. Interestingly, *Paracercomonas*, a genus that contains the predator isolate *Paracercomonas* CWPL, averaged 1.4% of the positive correlations with *Legionella* (Figure 1a) (Amaro et al. 2015). Both 16S rRNA sequencing and serogroup testing detected a large abundance spike for Legionella in July; however, a second smaller spike in April was only detected by 16S rRNA sequencing (Figure 1a). Additionally, beta diversity for the subsetted protist community (*Amoebozoa*, *Cercozoa*, *Ciliophora* and *Heteroloosea*) had a significant positive correlation with a model that accounted for both temperature and turbidity (*r* = 0.483). Bacterial beta diversity had the highest correlation with turbidity (*r* = 0.500). Specifics for each of the three‐time partitions are described below.

### 3.2 March/April: *Legionella* Increase Correlates With *Acanthamoeba* Hosts

**FIGURE 2:**
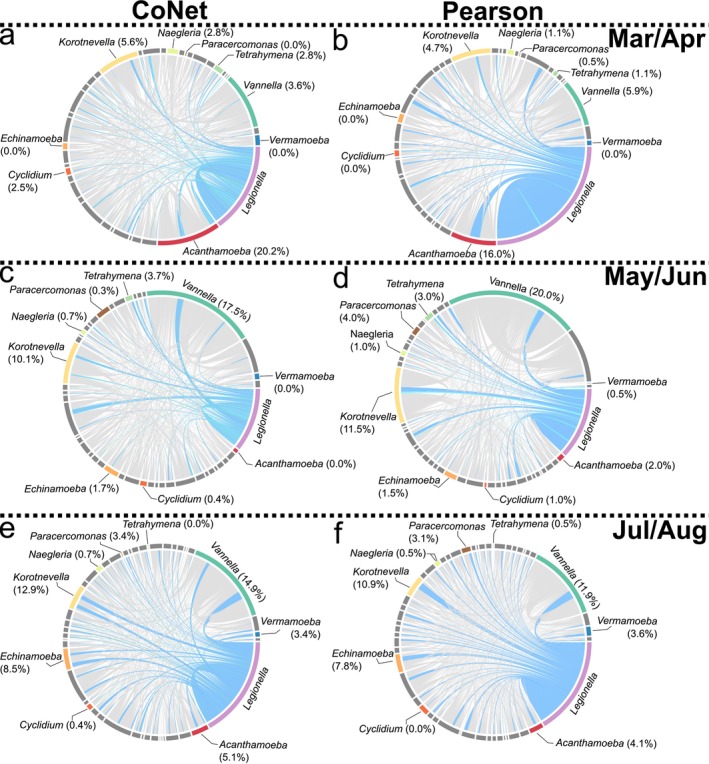
Genera *Legionella* was correlated with change through the seasons. Chord diagrams of CoNet (a, c, e) and Pearson (b, d, f) networks of protists. The ring is split into genera with genera that have been previously identified to interact with *Legionella* being coloured. *Korotnevella* was also highlighted due the comparable number of correlations between *Korotnevella* and *Legionella* compared to the protist hosts that had the most correlations with *Legionella*. Proportion of positive correlations with *Legionella* is indicated next to the genera that are coloured. All other genera are in grey. (a, b) The networks for March and April. (c, d) The networks for May and June. (e, f) The networks for July and August. Chords indicate strength of correlation. Blue > 0.7; 0.7 > light blue > 0.5; Green < 0.5; Red < 0.0. Grey indicates no correlation with *Legionella*. All correlations have a *p*‐value of < 0.05.

For the months of March and April, *Legionella* had its first peak in relative abundance (April), where it had the most correlations with *Acanthamoeba*. As mentioned above, only 16S rRNA profiling detected this peak. However, there was a relatively small decrease in serogroup density from March (1.12 × 10^6^ cells/L) to April (18.82 × 10^5^ cells/L). Despite this, *Legionella* ASVs during March had their lowest relative abundance (0.03%) for the entire 6‐month sampling period (Figure 1a), which then increased tenfold in April (0.43%). During March, *Acanthamoeba* (14.9%) was the prominent protist host in the community, with *Naegleria* (2.5%) being the second most common (Figure 1a). In April, *Acanthamoeba*'s average relative abundance decreased slightly (11.4%) and *Naegleria*'s increased to 25.0%, the highest relative proportion observed for any host genus during the study. Despite this high proportion, *Naegleria* had the fewest number of ASVs correlated with *Legionella* during March/April, whereas *Acanthamoeba* had the most (Figure 2a,b). Specifically, 20.2% (CoNet) and 16.0% (Pearson) of the significant *Legionella*‐protist correlations were with *Acanthamoeba*. Comparatively, *Naegleria* had less than 2% of the correlations in each network (Figure 2a,b). The third most relatively abundant protist host was *Vermamoeba* (1.7%), which also saw an increase in relative abundance during April (6.9%) (Figure 1a) but made up less than 2% of the *Legionella*‐protist connections. *Cyclidium*, *Vannella* and *Tetrahymena*, which were previously less than 1% of the population in March, increased to 2.9%, 2.8% and 1.7% of the community respectively by April. All other potential protist hosts had less than 1% relative abundance and all potential host genera besides *Acanthamoeba* had less than 6% of the connections with *Legionella* in March/April (Figure 1a). *Paracercomonas*, a predator genus, decreased in relative abundance from March (2.5%) to less than 1% of the community in April. *Paracercomonas* also had a negative correlation with *Legionella* in the CoNet network (Figure 2a).

### 3.3 May/June: *Legionella* Decline and Subsequent Increase Correlates With *Vannella* and *Korotnevella* Hosts

**FIGURE 3:**
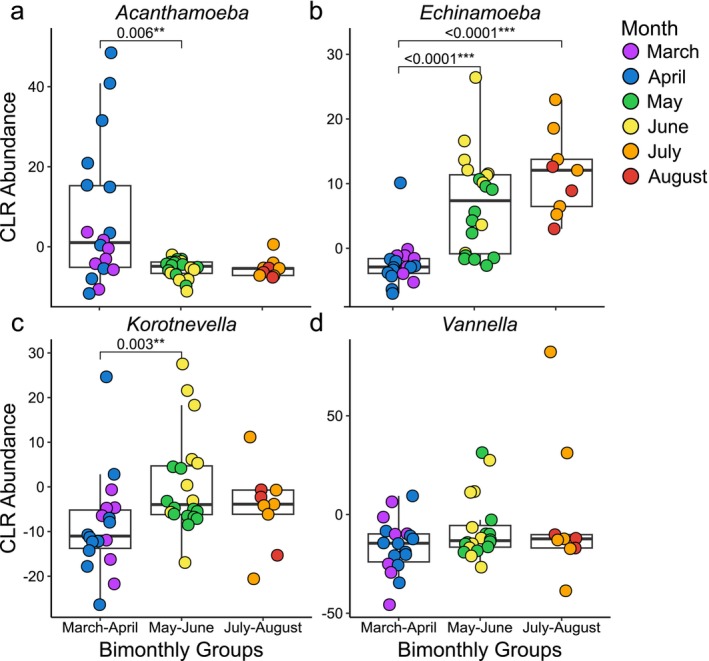
Log‐transformed abundances of genera that had substantial correlation with *Legionella* across the seasons. Bimonthly boxplots show changes in log‐transformed abundance for each genus. Dots are coloured by month. (a) *Acanthamoeba*, (b) *Echinamoeba*, (c) *Korotnevella* and (d) *Vannella*. Only statistically significant differences after FDR correction are shown (*α* < 0.05).

During the transition from April to May, the abundances for both *Legionella* and *Acanthamoeba* decreased dramatically (falling below 1%), with *Acanthamoeba* remaining at this level for the remainder of the study period (through August) (Figures 1a and 3a). Both the *Legionella* and *Acanthamoeba* decreases were shown to be significant by log transformed abundance (Figures 3a and S3a). Correspondingly, after *Acanthamoeba*'s population diminished, correlations with *Legionella* also became minimal, making up less than 3% of the total (Figures 1a and 2c,d). Although *Legionella*'s relative abundance diminished in May, it subsequently increased during June (0.29%). Similarly, the genera *Korotnevella* and *Tetrahymena* also increased in relative abundance during this two‐month period, showing increased correlations with *Legionella* (Figures 1a and 2).

**FIGURE 4:**
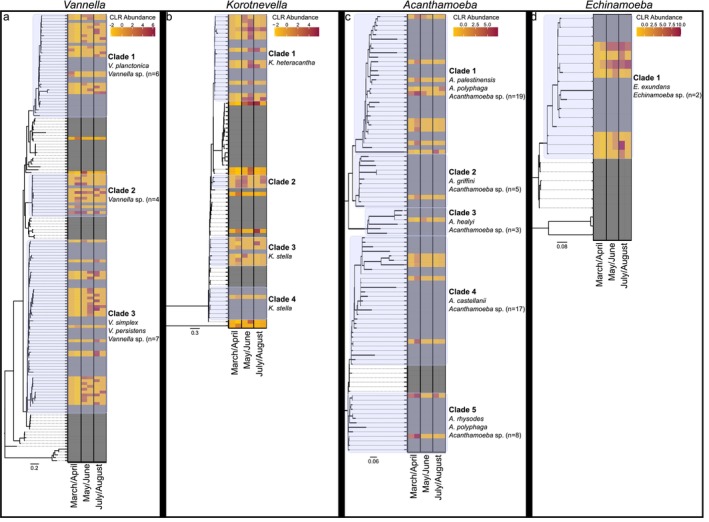
Phylogenies showing monthly change in log‐transformed relative abundance of each ASV per month for specified host genera. (a) *Vannella*, (b) *Korotnevella*, (c) *Acanthamoeba* and (d) *Echinamoeba*. The number of undefined species in a clade is indicated by *n*.

Notably, the frequency of *Vannella* decreased in May (< 1%) and increased in June (2.8%) mirroring the abundance pattern for *Legionella*. Correspondingly, *Vannella* was the protist with the most correlations with *Legionella* during this period, which constituted between 17% and 20% of the correlations with *Legionella* in each network (Figures 1a and 2c,d). The phylogenetic analyses showing the frequency distribution of species and lineages within each host genus through the seasons delineated three major lineages for *Vannella* ASVs (Figure 4a). One lineage (Clade 3, a *Vannella simplex* and *Vannella persistens* complex) was notable as it showed a dramatic increase in abundance during May/June and may have been responsible for the overall *Vannella* increase observed over this two‐month period.

For *Korotnevella*, we observed an overall increase in relative abundance during June to become 2.0% of the community (Figures 1a and 3c) and the log transformed abundance showed a significant increase from the first (March/April) to the second (May/June) time period (Figures 1a and 3c). Similar to *Vannella*, *Korotnevella* possessed an ASV lineage (Clade 1, *Korotnevella heteracantha*) that first appeared in May and then remained through the rest of the study period, which coincided with an increase in correlation between *Legionella* and *Korotnevella*, with 10.1% and 11.5% in the CoNet and Pearson networks, respectively (Figures 1a and 4b), again suggesting that a single lineage was primarily responsible for the overall pattern observed.

Two other protists had more than 1% of the *Legionella* correlations, *Tetrahymena* and *Echinamoeba*. *Tetrahymena* increased in relative abundance during May (14.4%) and decreased slightly in June (9.9%); however, there was no significant change in log transformed abundance from March/April to May/June. Despite this, there were increased correlations between *Tetrahymena* and *Legionella*, 2.8%–3.7% and 1.1%–3.0% for the CoNet and Pearson networks, respectively. Lastly, *Echinamoeba* also increased in relative abundance during May (1.2%) and June (5.7%) and showed a corresponding significant increase in log transformed abundance from March/April to May/June (Figures 1a and 3b). Correspondingly, the number of significant correlations between *Echinamoeba* and *Legionella* increased to 1.7% in the CoNet network and 1.5% for the Pearson network from the previous months where there were zero correlations (Figures 1a and 2c,d). However, the overall number of correlations for *Echinamoeba* and *Tetrahymena* was considerably lower than that for *Vannella* and *Korotnevella* (Figures 1a and 2c,d). Interestingly, *Paracercomonas*, despite being less than 1% of the population and being a putative predator of *Legionella*, shared 4% of the positive correlations with *Legionella* in the Pearson network (Figures 1a and 2d).

### 3.4 July/August: Large *Legionella* Increase to Unsafe Levels Correlates With *Vannella*, *Korotnevella* and *Echinamoeba* Hosts

During the July/August period, *Legionella* had its highest relative abundance for any month (July, 0.66%) (Figure 1a). July also had the highest density of *L. pneumophila* combined serogroups (1.52 × 10^7^ cells/L), which resulted in a red classification for the tower and treatment with ChemTreat CL‐49. *L. pneumophila* SG 4 dominated the serogroup distribution in July (Figure S5). Following treatment, August saw the tower return to a green serotype density (2.43 × 10^5^ cells/L) with a corresponding sharp decrease in *Legionella* ASV relative abundance (0.0071%) (Figure 1a). From June to July, the relative abundance of *Echinamoeba* increased from 5.7% of the community to 16.4% (Figure 1a) and there was a significant increase in log transformed abundance from March/April to July/August (Figure 3b). With the exception of *Echinamoeba* (16.4%), August saw a decline for all hosts, with them all dropping below 2% relative abundance (Figure 1a).

During July and August, despite their decline, *Vannella* and *Korotnevella* still showed the most correlations with *Legionella* (14.9% and 12.9% in the CoNet networks and 11.9% and 10.9% in the Pearson networks, respectively). The *Vannella* clade that increased dramatically in May/June (Clade 3, a *V. simplex* and *V. persistens* complex) remained present in July/August. The strong correlation between *Vannella* and *Legionella* in the following months may be attributed to a shift in *Vannella* lineages (Figure 4a). The remaining *Legionella*‐host correlations generally decreased. The main exception was *Echinamoeba*, which increased 8.5% in the CoNet network and 7.8% in the Pearson network (Figures 1a and 2e,f).

### 3.5 Microbial‐Environmental Correlations

To understand the relationship among environmental factors, protists and *Legionella*, we performed correlation analysis between *Legionella* and protists with environmental factors. Previous testing at the Savannah River Site demonstrated a correlation between chlorine and DO concentrations with *Legionella pneumophila* (Brigmon et al. 2020). We observed that no environmental factors were correlated with combined *Legionella* ASVs, but there were environmental correlations with *L. pneumophila* SGs (Table S4). DO (*r* = −0.59) and pH (*r* = −0.66) were both negatively correlated with the combined *L. pneumophila* SGs. Turbidity (*r* = 0.76), Br (*r* = 0.42) and Cl (*r* = 0.42) were positively correlated with combined *L. pneumophila* SGs and three of the four SGs separately (SG 1, 4 and 6). While there was no correlation between combined SGs and temperature, there was a positive correlation between SG2 and temperature (*r* = 0.78).

With the exception of *Tetrahymena* and *Vannella*, potential host genera were also correlated with environmental parameters. *Acanthamoeba* (*r* = −0.48) and *Naegleria* (*r* = −0.38) had a negative correlation with temperature, while *Echinamoeba* (*r* = 0.78) and *Korotnevella* (*r* = 0.49) had a positive correlation (Table S5). *Echinamoeba* had a negative correlation with DO (*r* = −0.46). *Acanthamoeba* also had a negative correlation with pH (*r* = −0.50) and a positive correlation with turbidity (*r* = 0.42).

Turbidity had the highest correlation with bacterial beta diversity (*r* = 0.50) (Table S6). Comparatively, temperature had the highest correlation for eukaryotic beta diversity (*r* = 0.29) (Table S6). Temperature had the highest correlation for bacterial Shannon (alpha) diversity (*r* = 0.68). For eukaryotic Shannon diversity, there were no significant correlations between it and environmental factors.

## 4 Discussion

During the 6 months of this study, we found evidence that *Legionella* switched hosts through the seasons. We observed shifts in the relative abundance for *Legionella* that mirrored shifts in the relative abundance for three protist genera known to contain host species (*Acanthamoeba*, *Echinamoeba* and *Vannella*) as well as a single protist genus (*Korotnevella*) that had substantial correlation with *Legionella* yet contained no known host species. Possible factors affecting host availability include environmental changes and *Legionella* host killing. However, we did not observe any cyclical fluctuations in the host protist populations, as their frequency did not consistently rise in the absence of *Legionella* and fall when *Legionella* populations increased, which would have supported *Legionella* host killing. Rather, through the seasons, different host genera correlated with environmental parameters whereas *Legionella* did not, suggesting that shifts in the taxonomic composition of the protist community were driven by the environment. This study was limited by sampling from a single water‐cooling tower, which may restrict the broader applicability of these findings to other water‐cooling towers and engineered water systems. Additionally, both DFA and amplicon sequencing detect intact *Legionella* cells regardless of their viability, meaning that observed frequencies may include non‐viable cells. In contrast, inclusion of culture‐based methods would allow for the quantification of viable *Legionella* cells. Nevertheless, combined, these results suggest that the environment has a major influence on protist taxonomic temporal distribution, which in turn influences *Legionella* temporal abundance.

Overall, temperature was a major factor correlated with host occurrence through the two seasons. However, since our analysis was limited to one water‐cooling tower, the observed seasonal shifts in host occurrence may not apply to other towers, which could exhibit different host associations or temperature preferences. Nonetheless, *Acanthamoeba* served as host in the colder months of early spring during the first peak in *Legionella* abundance, followed by *Vannella* and *Korotnevella* in late spring–early summer, joined by *Echinamoeba* in mid‐summer, with these last three genera serving as hosts during the second peak in *Legionella* abundance. The work of Nielsen et al. (2014) supports cold tolerance for *Acanthamoeba* as they showed *Acanthamoeba castellanii* to replicate at temperatures as low as 18°C. Additional environmental factors may have contributed to the variation in host occurrence. For example, high pH has been shown to reduce *A. castellanii* 's ability to form resistant cysts (Aqeel et al. 2013) and, in addition to temperature, May saw a strong rise in pH, which may have contributed to the decline in *Acanthamoeba*. For *Echinamoeba* protists, a previous study showed their distribution within an industrial water system to be skewed towards low DO (Ramirez et al. 2014) and the peak in *Echinamoeba* occurrence detected here coincided with a strong decrease in DO during the summer. Additionally, the biocide shocking in the summer and other factors not captured in this study (e.g., inclement weather and dissolved organic carbon) could have influenced microbial dynamics and contributed to the patterns observed in this study. The contrast in taxonomy and diversity of hosts available between the spring and summer peaks in *Legionella* abundance likely contributed to the different characteristics of each peak. Specifically, the finding that none of the *Legionella* serotypes monitored contributed to the spring peak suggests that the spring and summer peaks were taxonomically distinct, with this taxonomic distinction possibly resulting from the availability of only a single host genus in the spring that additionally limited host suitability among *Legionella* lineages. In contrast, the availability of three host genera in the summer likely increased the diversity of host suitability among *Legionella* lineages and may have contributed to the more intense summer peak.

Previous *Legionella* laboratory studies have shown a diversity of host infection and intra‐host growth characteristics. For example, studies have shown (i) shifts in temperature to modulate *Legionella* growth within hosts (Barbaree et al. 1986; Fields et al. 1984), (ii) both intra‐ and inter‐species variation in ability to infect and replicate within different protist hosts (Dey et al. 2009; Neumeister et al. 1997) and (iii) intraspecific growth variation within the same host (Hasni et al. 2020). *Legionella* secretes a wide range of effector proteins into host cells to facilitate infection and intracellular replication, and this high protein diversity is likely an important factor contributing to the diversity of intra‐ and interspecies abilities to infect and grow within different protists. For example, over 18,000 effector proteins have been predicted for 58 *Legionella* species, with only eight core effectors (Gomez‐Valero et al. 2019). Of these eight, two were at least partially required for growth in *A. castellanii* (Burstein et al. 2016). The function for the other six remained unknown; however, it could involve growth in other hosts (Burstein et al. 2016; Gomez‐Valero et al. 2019). In addition to effector‐mediated infection and replication, quorum sensing may contribute to *Legionella* virulence. A crucial gene involved in *Legionella* quorum sensing (lqsA) was found in only 19 of the 58 *Legionella* genomes analysed by Herran et al. (2021). lqsA encodes for an enzyme that synthesises the autoinducer LAI‐1 which has demonstrated an ability to promote bacterial uptake by *A. castellanii* , potentially through recognition by a sensor kinase (LqsS), which promotes host‐pathogen interactions through a phosphorylation cascade (Tiaden et al. 2010). The diversity of *Legionella*'s effector proteins signifies the extended co‐evolution with protist hosts, reflecting *Legionella*'s ongoing evolution to evade lysosomal degradation in a variety of protists (Shames 2023).

Previous laboratory studies have also shown strong differences in host susceptibility both among and within genera (Declerck et al. 2005; Tyndall and Domingue 1982). Intra‐genus differences in *Vannella* could explain the strong correlation between *Vannella* and *Legionella* in the later months after *Vannella* Clade 3 appeared. This suggests that changes at the species or lineage level, rather than the genus level, may underlie *Vannella*'s association with *Legionella*. Differences in susceptibility could be influenced by different mechanisms of uptake. For example, different sugar‐binding receptors for attachment and receptor‐mediated endocytosis of *Legionella* have been observed in different protists. Specifically, in *A. castellanii* , *L. pneumophila* had high binding affinity for mannose‐binding receptors (Declerck, Behets, De Keersmaecker, et al. 2007; Harb et al. 1998); whereas, in *N. lovaniensis* , *L. pneumophila* had high binding affinity for N‐acetyl‐d‐galactosamine receptors (Declerck, Behets, De Keersmaecker, et al. 2007; Harb et al. 1998). Additionally, differences in tyrosine dephosphorylation have been detected during uptake of *Legionella* by *A. polyphaga* and *V. vermiformis* . Attachment of *Legionella* to *V. vermiformis* was associated with tyrosine dephosphorylation of multiple host proteins, while attachment to *A. polyphaga* had only a slight dephosphorylation of a single protein (Declerck, Behets, De Keersmaecker, et al. 2007; Harb et al. 1998).

## 5 Conclusion

The longitudinal study presented here suggests that *Legionella* frequency can be modulated by temperature‐dependent protist availability. In addition, we highlight a limitation of *Legionella* serotype testing when used for surveillance of man‐made water systems. Specifically, peaks in *Legionella* abundance can occur that do not involve *L. pneumophila* serotypes one, two, four and six. Moving forward, amplicon sequencing or Q‐PCR‐based approaches might provide viable alternatives in critical circumstances. Furthermore, continued investigation of potential *Legionella* hosts and how they are impacted by environmental dynamics and biocontrol applications provides another potential avenue for Legionnaires' disease outbreak prediction or prevention. For example, protists such as those identified here could be targeted in novel prediction/prevention strategies. Understanding how *Legionella* persists in water systems will advance our understanding of the pathology of Legionnaires' disease, such as the impact of the adaptability of *Legionella* to dynamic environments with changing protist communities.

## Author Contributions

**Suzanne Crull:** investigation, formal analysis, methodology, software, visualization, writing – review and editing, writing – original draft. **Emlyn Hammer:** investigation, methodology, formal analysis, writing – review and editing. **Allison E. Mann:** investigation, writing – review and editing. **Lauren M. O'Connell:** investigation, writing – review and editing. **Ashlyn Soule:** investigation. **Elizabeth Griffith:** investigation. **Thomas Blouin:** investigation, writing – review and editing. **Robin L. Brigmon:** conceptualization, investigation, formal analysis, resources, writing – review and editing. **Vincent P. Richards:** funding acquisition, resources, supervision, conceptualization, writing – review and editing.

## Conflicts of Interest

The authors declare no conflicts of interest.

## Supporting information


**Data S1.** Supplementary figures.

**Data S2.** Supplementary tables.

## Data Availability

The datasets generated and analysed during the current study are available in the European Nucleotide Archive repository under accession number PRJEB84035. All preprocessing and analysis scripts are available at https://github.com/sacrull/Legionella.
