# Supplementary material for: Seasonal Host Shifts for Legionella Within an Industrial Water‐Cooling System
Source: Environ Microbiol Rep. 2025 Jun 30;17(4):e70132. doi: 10.1111/1758-2229.70132 (PMC12207018; doi:10.1111/1758-2229.70132)
Supplement: Supplementary file 1 — Data S1. Supplementary figures. [file EMI4-17-e70132-s001.docx]

Figure S1: Phylum-level relative abundance plots for the eukaryotic community (18S rRNA). (a) Eukaryotic community by month. (b) Eukaryotic community by sample.

Figure S2: Rarefaction curves generated from quality filtered data for (a) bacterial and (b) eukaryotic communities and colored by month.

Figure S3: Differences in center-log transformed abundance of *Legionella* through the seasons. (a) Bimonthly differences in CLR abundance. (b) Monthly differences in CLR abundance. Dots are colored by month. Only statistically significant differences after FDR correction are shown (⍺ ≤ 0.05).

Figure S4: *Legionella* and protist genera average relative abundance by sample. *Legionella* is represented by the blue line overlayed on the bar graph. In the bar chart, protist genera possessing species known to interact with Legionella are colored. *Korotnevella* was also colored due the comparable number of correlations with Legionella compared to the protist hosts with the most correlations. All other genera belonging to Amoebozoa, Cercozoa, Ciliophora, and Heterolobosea are colored gray.

Figure S5: Concentration (log scaled) of individual *L. pneumophila* serogroups by month.
